# Unraveling the Complexity: A Rare Case of Systemic Sclerosis, Rheumatoid Arthritis, and Systemic Lupus Erythematosus Overlap Syndrome

**DOI:** 10.7759/cureus.68405

**Published:** 2024-09-01

**Authors:** Sava Nanda Gopal, Deepthi Vakati, Kanimozhi David, Saranya Palanisamy, Kannan Rajendran

**Affiliations:** 1 Internal Medicine, Saveetha Medical College and Hospitals, Saveetha Institute of Medical and Technical Sciences, Saveetha University, Chennai, IND

**Keywords:** autoimmune diseases, overlap syndrome, rheumatoid arthritis, systemic lupus erythematosus, systemic sclerosis

## Abstract

Overlap syndromes involving systemic sclerosis (SSc), rheumatoid arthritis (RA), and systemic lupus erythematosus (SLE) are rare and present significant diagnostic and therapeutic challenges. This case report details a 54-year-old female who presented with a constellation of clinical and serological features suggestive of both SSc, RA, and SLE. The patient's clinical course, diagnostic findings, and response to treatment are discussed highlighting the need for a multidisciplinary approach in managing such complex cases.

## Introduction

Three different autoimmune diseases, systemic sclerosis (SSc), rheumatoid arthritis (RA), and systemic lupus erythematosus (SLE), have immunological dysregulation, multisystem involvement, and chronic inflammation in common [1]. In a subgroup of people, these illnesses can coexist while having different pathophysiologies and clinical presentations creating an overlap syndrome. Since each condition's symptoms and indications manifest simultaneously, this overlap creates a unique clinical challenge. [2].

Overlap syndromes are an uncommon but well-known subset of autoimmune disorders. When SSc, RA, and SLE coexist in one patient, it can make diagnosis and treatment planning more difficult. RA primarily affects the joints resulting in erosive arthritis; SLE is a systemic condition with a wide range of manifestations including in the skin, joints, kidneys, and the central nervous system; and SSc is primarily characterized by skin thickening and fibrosis as well as involvement of internal organs [3].

The combination of these illnesses may provide an unusual clinical history with overlapping symptoms that could make it difficult to distinguish the distinct roles played by each disease. Because of this overlap, diagnosing and treating these conditions requires a multidisciplinary approach, as the approaches used to manage one ailment may affect the others [4]. Moreover, the presence of multiple autoimmune conditions often correlates with a more severe disease course and a greater risk of complications [5].

In this report, we present the case of a 54-year-old female with an overlap syndrome involving SSc, RA, and SLE. The case highlights the complexity of diagnosing and managing patients with multiple autoimmune diseases and underscores the importance of a comprehensive and individualized treatment approach.

## Case presentation

**Figure 1:**
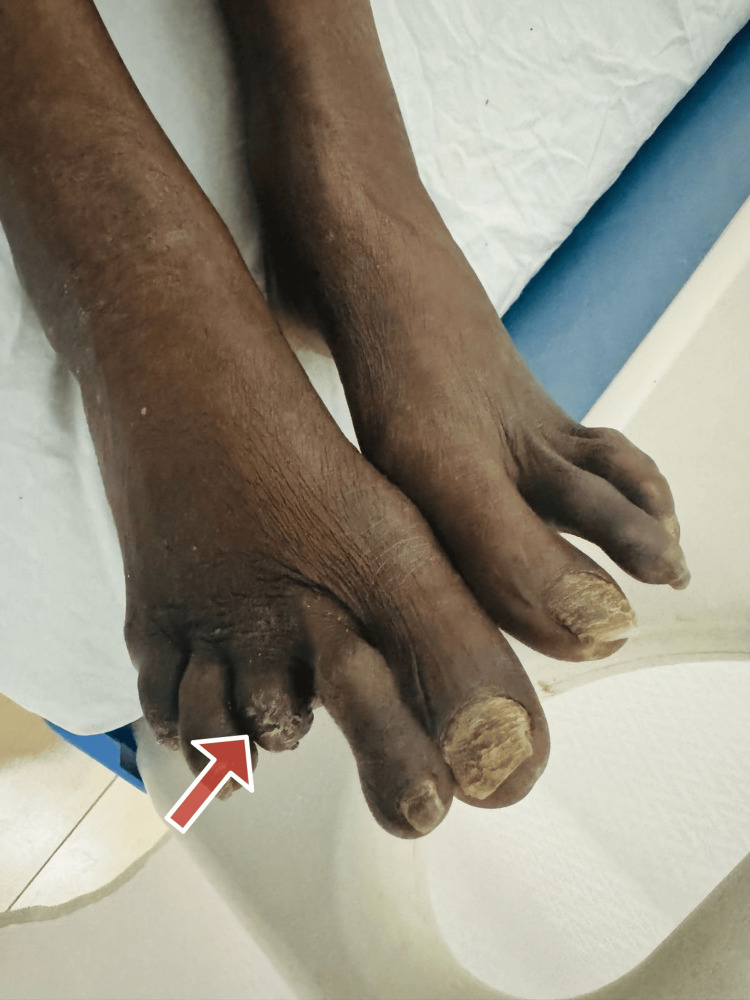
Autoamputation of the right third metatarsal This image depicts autoamputation in a patient with systemic sclerosis. The red arrow points to the affected finger which has undergone spontaneous amputation due to severe tissue damage caused by the disease

**Figure 2:**
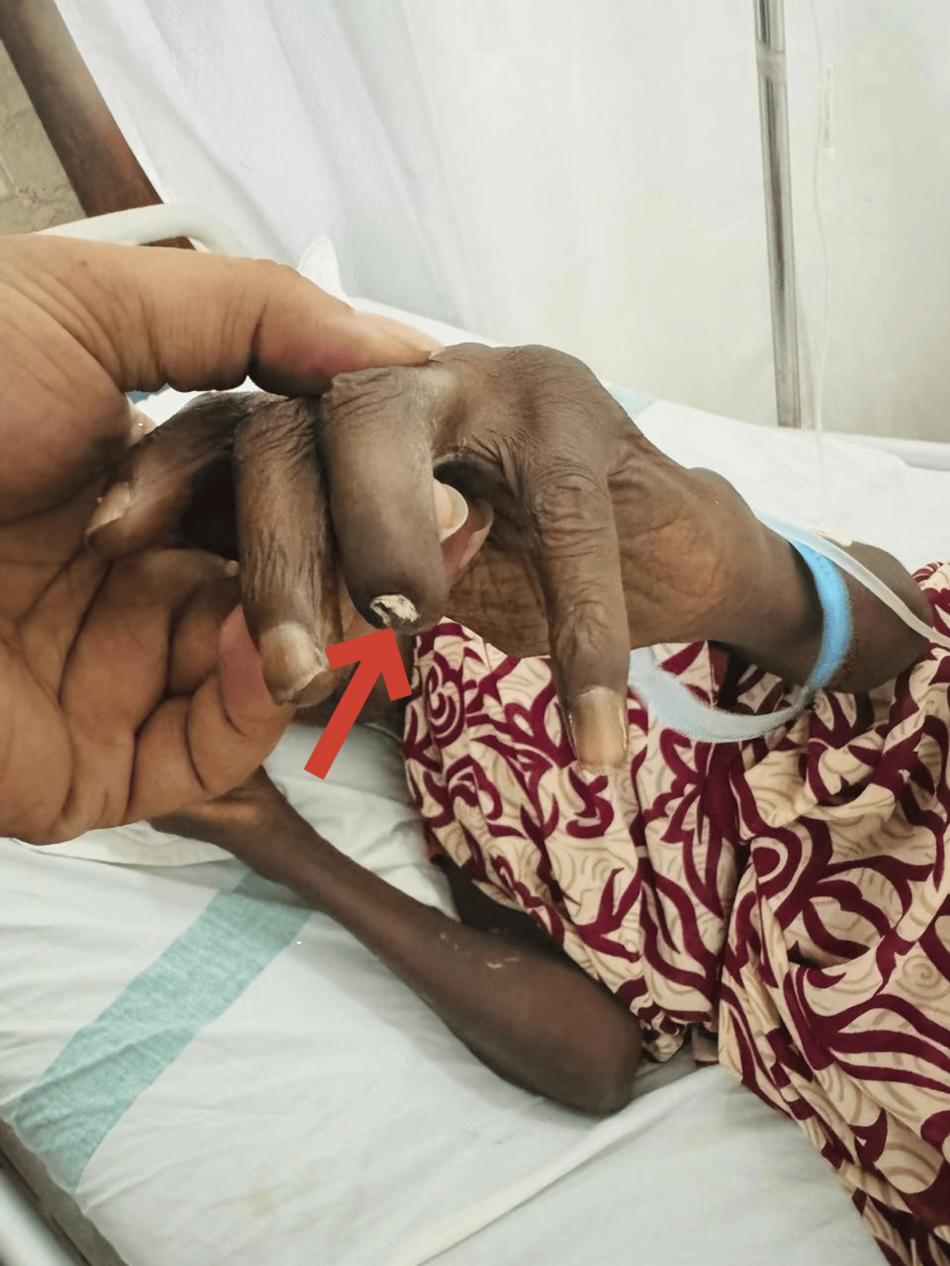
Autoamputation seen in the left fourth distal phalanx This image depicts autoamputation in a patient with systemic sclerosis. The red arrow points to the affected finger which has undergone spontaneous amputation due to severe tissue damage caused by the disease

A 54-year-old woman from South India presented to our hospital with complaints of progressive skin tightening, multiple joint pain, dyspnea, and abdominal pain over the previous six months. The patient also reported episodic facial rashes and photosensitivity. Upon arrival, her vitals were normal. General examination was normal, except for autoamputations seen in the right third metatarsal and left fourth finger as seen in Figures 1-2.

Clinical examination

**Figure 3:**
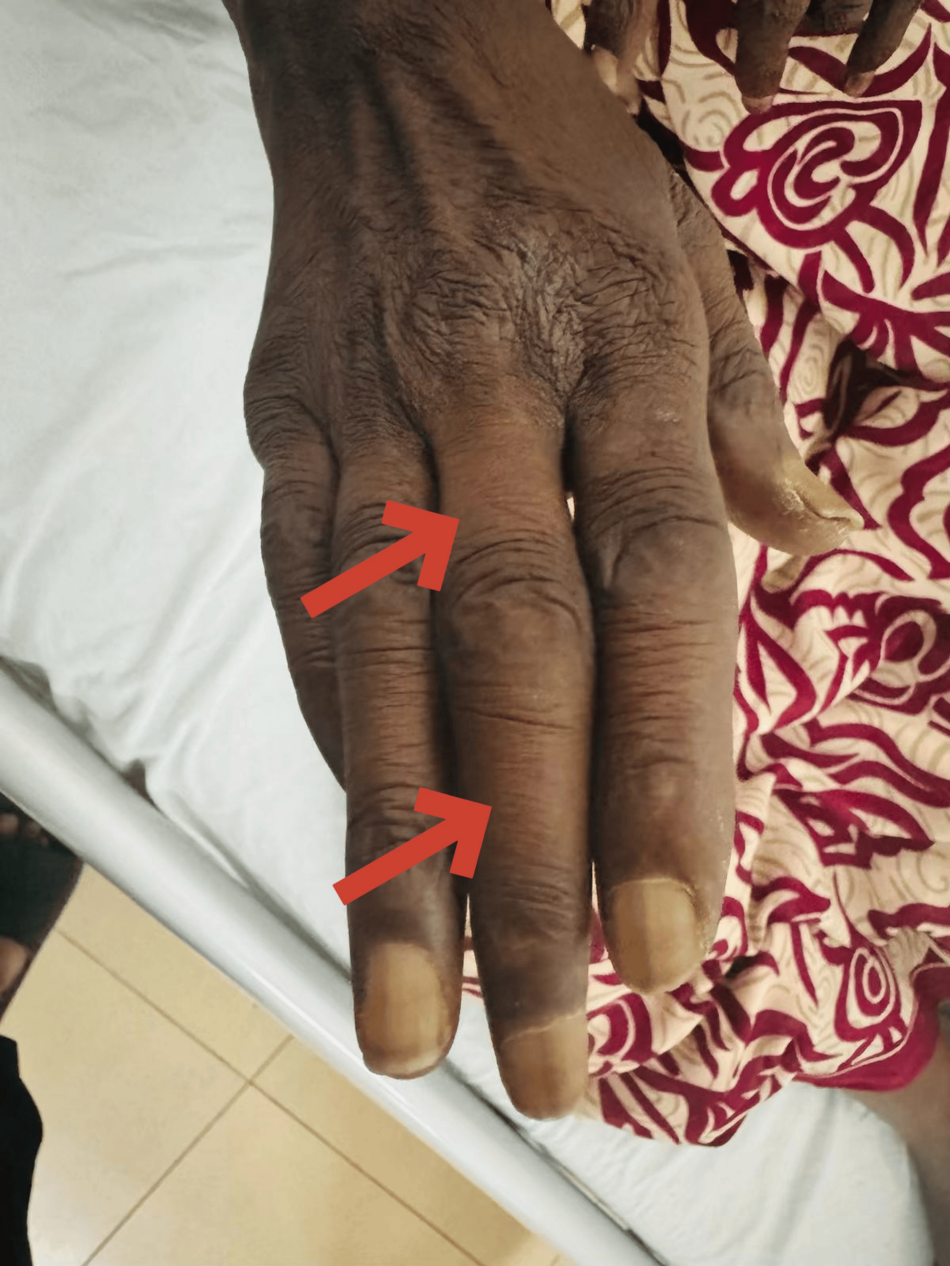
Thickening of the skin seen on the right third finger The image shows the hand of a patient with the second and third fingers exhibiting digital sclerosis indicated by arrows. The skin appears thickened and tightened characteristic of sclerodactyly, which is often seen in conditions such as systemic sclerosis

The patient presented with thickened skin over the hands and forearms as depicted in Figure 3.

**Figure 4:**
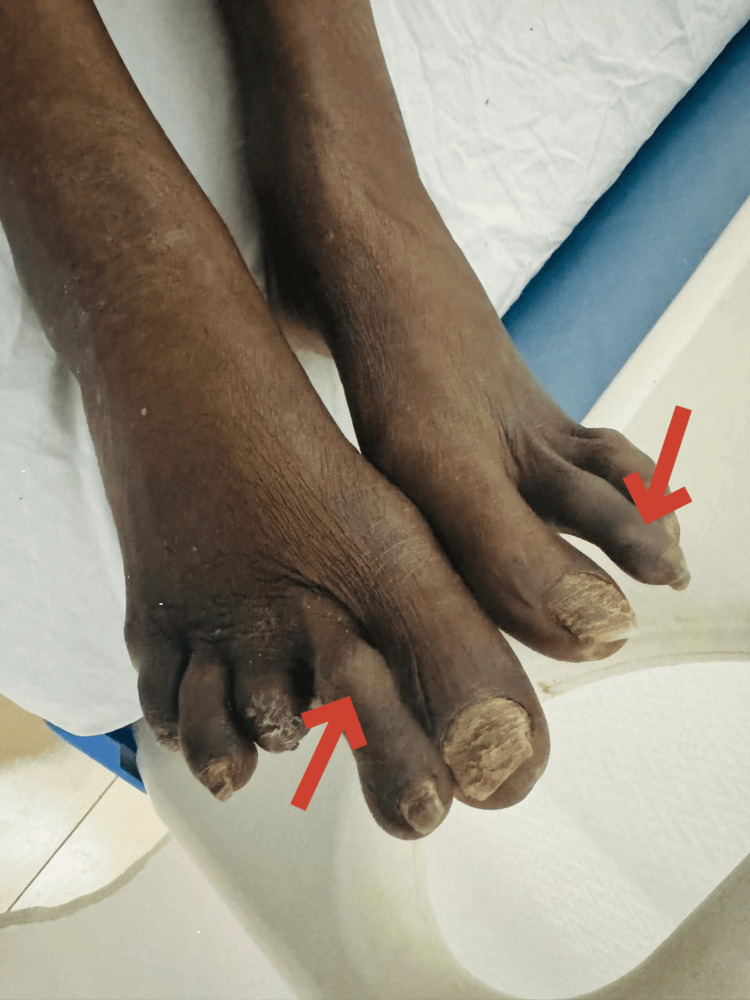
Finger swellings seen on the left and right second metatarsal bone This image shows the feet of a patient with rheumatoid arthritis (RA). The red arrows indicate swelling in the toes, a common symptom of RA

Swelling and tenderness were observed in the small joints of the foot, as illustrated in Figure 4. Additionally, lung auscultation revealed bibasilar crackles, and the patient reported worsening dyspnea on exertion.

Laboratory findings

Hemoglobin (Hb) levels were found to be 8.5 g/dl, which is below the normal range for females (12-15.5 g/dL). The RA factor was elevated at 100.17 IU/ml, exceeding the normal reference value of less than 20 IU/ml. Antinuclear antibody (ANA) tests revealed positive results for Mi-2, Ro-52, AMA-M2, and Histone-3, as detailed in Table 1.

**Table 1:** ANA profile of the patient ANA: Antinuclear antibody; SSA: Sjögren's-syndrome-related antigen A; SSB: Sjögren's syndrome type B; PCNA: proliferating cell nuclear antigen; AMA-M2: antimitochondrial antibody; DFS70: dense fine speckled 70

Autoantibody	Result	Significance
Mi-2	Positive (+++)	Associated with dermatomyositis. Indicates muscle weakness and characteristic skin rashes
Ku	Positive (+)	Seen in overlap syndromes, particularly in polymyositis and systemic sclerosis
RNP/Sm (RNP)	Negative	Absence of RNP antibodies. These are usually associated with mixed connective tissue disease
Sm	Negative	Absence of Sm antibodies, which are highly specific for systemic lupus erythematosus (SLE)
SS-A native (60kDa) (SSA)	Negative	Absence of SSA antibodies, often associated with Sjögren's syndrome and SLE
Ro-52 recombinant (Ro 52)	Positive (+++)	Can be associated with Sjögren's syndrome, systemic lupus erythematosus, and other autoimmune conditions
SS-B (SSB)	Negative	Absence of SSB antibodies, which are commonly associated with Sjögren's syndrome and SLE
Scl-70 (Scl)	Negative	Absence of Scl-70 antibodies, which are strongly associated with systemic sclerosis, especially diffuse cutaneous form
PM-Scl100 (PM100)	Negative	Absence of PM-Scl antibodies, which are associated with polymyositis and systemic sclerosis overlap syndrome
Jo-1 (Jo)	Negative	Absence of Jo-1 antibodies, commonly seen in polymyositis and interstitial lung disease (antisynthetase syndrome)
Centromere B (CB)	Negative	Absence of centromere B antibodies, which are associated with limited cutaneous systemic sclerosis (CREST syndrome)
PCNA	Negative	Absence of PCNA antibodies, associated with SLE
dsDNA (DNA)	Negative	Absence of dsDNA antibodies, which are highly specific for SLE
Nucleosomes (NUC)	Negative	Absence of nucleosome antibodies, seen in SLE and drug-induced lupus
Histone (HI)	Positive (+++)	Typically associated with drug-induced lupus erythematosus
Ribosomal protein (RIB)	Negative	Absence of ribosomal P antibodies, seen in neuropsychiatric lupus
AMA-M2	Positive (++)	Associated with primary biliary cholangitis (PBC)
DFS70	Negative	Absence of DFS70 antibodies, which are sometimes seen in healthy individuals and various autoimmune diseases

The anti-dsDNA test was positive and the anti-CCP antibody level was significantly elevated at 601.1 RU/ml, while the normal value for the anti-cyclic citrullinated peptide (anti-CCP) is less than 25 RU/ml indicating a high likelihood of rheumatoid arthritis. Additionally, the direct Coombs test returned a positive result as detailed in Table 2.

**Table 2:** Relevant immunological and hematological investigations Anti-CCP: Anti-cyclic citrullinated peptide

Test	Result	Reference range	Significance
Anti-dsDNA	Positive	Negative	Indicates high specificity for systemic lupus erythematosus (SLE)
Anti-CCP	601.1 RU/ml	<25 RU/ml-negative; >25 RU/ml-positive	Significantly elevated, highly suggestive of rheumatoid arthritis (RA)
Direct Coombs test	Positive	Negative	Indicates the presence of antibodies against red blood cells, often associated with autoimmune hemolyticanemia
Complement C3	52 mg/dl	88-165 mg/dl	Decreased C3 levels are common in active SLE, indicating complement consumption due to immune complex formation
Complement C4	9.8 mg/dl	14-44 mg/dl	Decreased C4 levels, also suggestive of active SLE, reflecting ongoing inflammation and immune activity

Radiological findings

**Figure 5:**
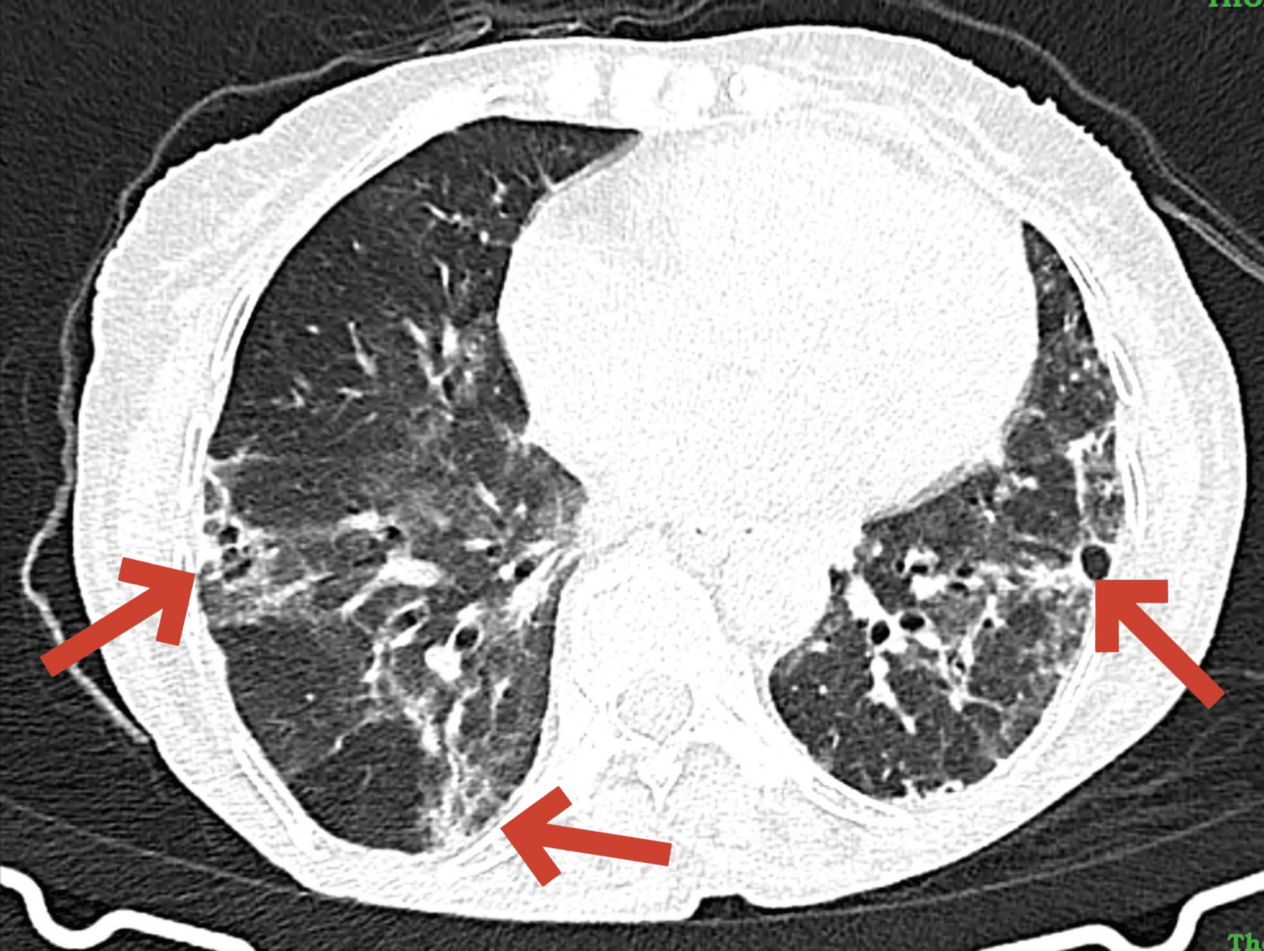
Coronal CT thorax showing lung involvement CT: Computed tomograhy The coronal CT image demonstrates significant lung involvement characterized by areas of ground-glass opacities and tractional bronchiectasis as indicated by the red arrows. These findings are consistent with interstitial lung disease likely secondary to the patient's overlap syndrome

**Figure 6:**
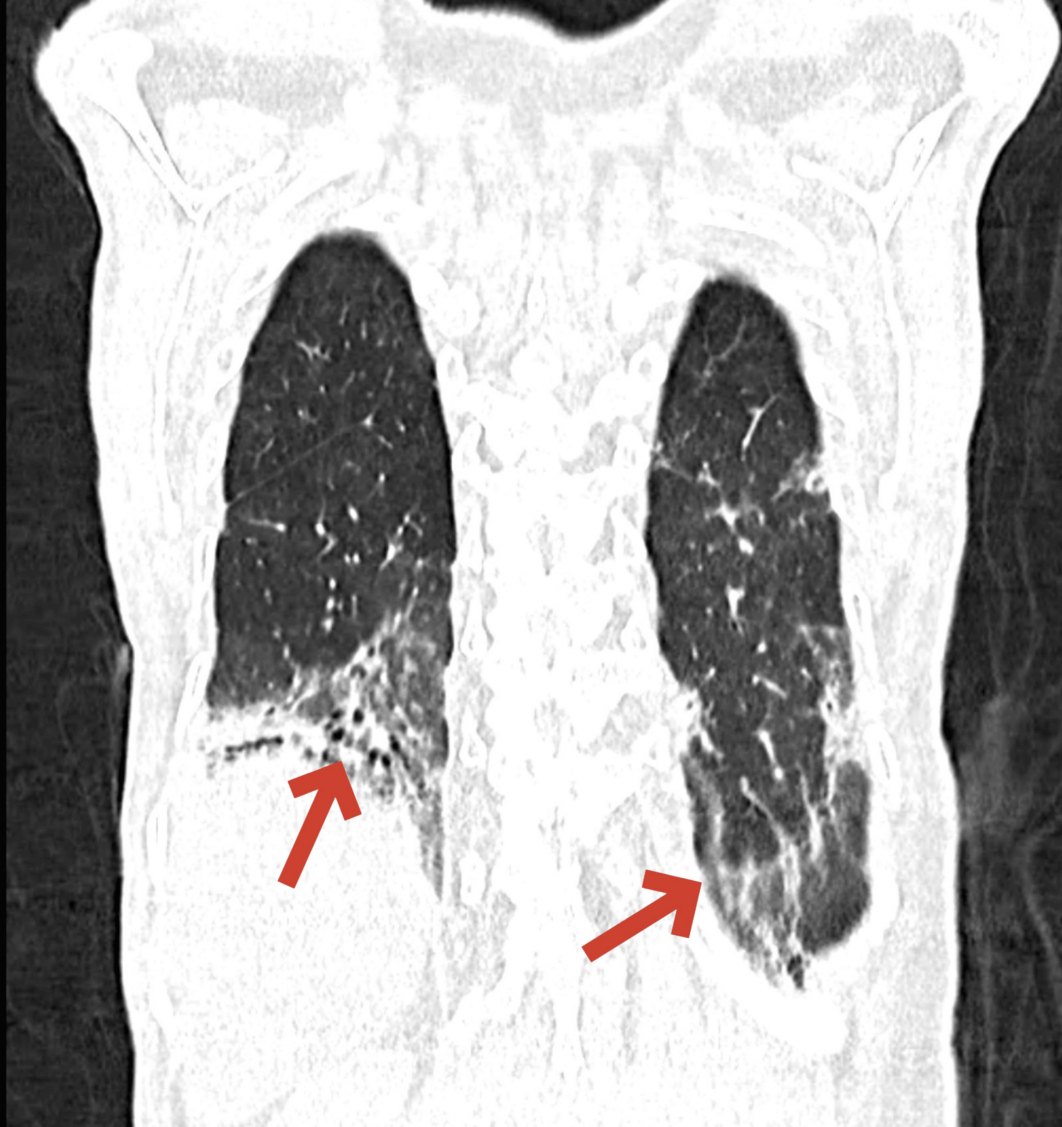
Axial CT thorax revealing diffuse lung pathology CT: Computed tomography The axial CT image reveals diffuse lung pathology with multifocal areas of ground-glass opacities, consolidatory changes, and tractional bronchiectasis as highlighted by the red arrows. These features suggest a progressive interstitial lung disease in the context of a systemic autoimmune disorder

A high-resolution CT scan of the chest showed evidence of interstitial lung disease with tractional bronchiectasis, multifocal areas of ground-glass opacities, and evolving consolidatory changes in bilateral lung fields, as shown in Figures 5-6.

Diagnosis

Based on the clinical findings and serological markers, a diagnosis of overlap syndrome involving SSc, RA, and SLE was made.

Treatment

After confirming the diagnosis, the patient was started on low-dose prednisolone, folic acid, methotrexate, and mycophenolate mofetil (MMF) to manage the interstitial lung disease and skin involvement. Hydroxychloroquine was prescribed to control lupus-specific symptoms. An angiotensin-converting enzyme (ACE) inhibitor was added to address Raynaud’s phenomenon and prevent potential renal involvement. Due to her anemia (Hb, 8.5 g/dl), she also received a blood transfusion to improve hemoglobin levels. The patient showed symptomatic improvement following the initiation of treatment and was discharged with the prescribed medications.

## Discussion

Because symptoms from three different autoimmune diseases SSc, RA, and SLE appear at the same time, this overlap syndrome poses serious diagnostic and treatment challenges [6]. The coexistence of these conditions can amplify the inflammatory response leading to a more severe disease course as seen in the case of the 54-year-old female patient who presented with interstitial lung disease, skin thickening, and joint involvement [7]. Diagnostic complexities arise from the variable serological profiles and overlapping clinical features necessitating a multidisciplinary approach for effective management [8]. The treatment regimen often includes a combination of immunosuppressants tailored to address the specific manifestations of each disease as seen in this case with methotrexate, MMF, and hydroxychloroquine [9]. Because of the increased disease burden associated with overlap syndromes, the prognosis is typically worse underscoring the importance of prompt and intensive management. To improve patient outcomes and manage the chronic nature of these illnesses, comprehensive care and routine follow-up are needed [10]. More research is needed to identify tailored treatments for these complicated situations and to gain a deeper understanding of the pathophysiology of overlap syndromes.

## Conclusions

This case report highlights the importance of identifying overlap syndromes in patients exhibiting mixed characteristics of autoimmune disorders. Improving patient outcomes requires early diagnosis and an interdisciplinary approach to management. Further studies are necessary to determine the etiology and best practices for treating these uncommon overlap disorders.
